# CORRELATES BETWEEN SENSORY IMPAIRMENTS AND PAIN CHARACTERISTICS IN MEDICARE BENEFICIARIES

**DOI:** 10.1093/geroni/igae098.1402

**Published:** 2024-12-31

**Authors:** Christina Mu, Alisha Thompson, Bailee Brekke, Safiyyah Okoye, Lama Assi, Sahar Assi, Soomi Lee

**Affiliations:** University of California, San Francisco, San Francisco, California, United States; Louisiana State University, Baton Rouge, Louisiana, United States; Miami University, W. College Corner, Indiana, United States; Drexel University, Philadelphia, Pennsylvania, United States; Louisiana State University Health Center, New Orleans, Louisiana, United States; Johns Hopkins University, Baltimore, Maryland, United States; The Pennsylvania State University, University Park, Pennsylvania, United States

## Abstract

Objective. Sensory impairments (SI) are associated with comorbidities, functional difficulties, and poor self-rated health. Few studies have examined associations between SI and pain, which is a strong correlate of poor health in later life. We examined the associations between SI and pain. Methods. We used cross-sectional data from the 2021 National Health and Aging Trends Study (n=2979 community-living older adults; ages ≥ 65 years, 57% ages 75-84). We included four measures of SI: objective hearing loss (HL), measured as pure-tone average >25 dB HL; subjective HL, measured as hearing aid use or being unable to hear in certain situations; objective vision loss (VL), measured as logMAR >0.30 for distance or near vision or logCS <1.55 for contrast sensitivity; and subjective VL, measured as inability to see in certain situations. We examined four participant-reported outcomes: being bothered by pain; activity-limiting pain; use of pain medication; and pain locations over the past month. Separate logistic and linear regressions controlled for sociodemographic and health characteristics (e.g., depressive symptoms, chronic conditions). Results. Objective HL was associated with higher odds of activity-limiting pain (aOR=1.33, 95%CI=1.02-1.74). Subjective VL was associated with higher odds of activity-limiting pain (aOR=1.44, 95%CI=1.07-1.93), lower odds of using pain medication (aOR=0.70, 95%CI=0.54-0.92), and more pain locations (B=0.59, 95%CI=0.14-0.97). Compared to having no SI, reporting one or more subjective SI was associated with more pain locations (single SI: B=0.23, 95%CI=0.006-0.46; dual SI: B=0.73, 95%CI=0.08-1.39). Conclusion. Findings suggest SI and pain are related, which may have important implications in clinical settings and research.

